# Patterns of Hepatitis C Virus RNA Levels during Acute Infection: The InC^3^ Study

**DOI:** 10.1371/journal.pone.0122232

**Published:** 2015-04-02

**Authors:** Behzad Hajarizadeh, Bart Grady, Kimberly Page, Arthur Y. Kim, Barbara H. McGovern, Andrea L. Cox, Thomas M. Rice, Rachel Sacks-Davis, Julie Bruneau, Meghan Morris, Janaki Amin, Janke Schinkel, Tanya Applegate, Lisa Maher, Margaret Hellard, Andrew R. Lloyd, Maria Prins, Gregory J. Dore, Jason Grebely, InC Study Group

**Affiliations:** 1 The Kirby Institute, UNSW Australia (University of New South Wales), Sydney, NSW, Australia; 2 Cluster Infectious Diseases, GGD Public Health Service of Amsterdam, Amsterdam, The Netherlands; 3 Department of Epidemiology and Biostatistics, University of California, San Francisco, San Francisco, California, United States of America; 4 Harvard Medical School, Boston, Massachusetts, United States of America; 5 Tufts Medical School, Boston, Massachusetts, United States of America; 6 Abbvie, Chicago, Illinois, United States of America; 7 Department of Medicine, Johns Hopkins Medical Institutions, Baltimore, Maryland, United States of America; 8 Burnet Institute, Melbourne, VIC, Australia; 9 Department of Epidemiology and Preventive Medicine, Monash University, Melbourne, Australia; 10 CRCHUM, Université de Montréal, Montreal, QC, Canada; 11 Academic Medical Center, Amsterdam, The Netherlands; 12 Inflammation and Infection Research Centre, School of Medical Sciences, UNSW Australia, Sydney, NSW, Australia; University of Cincinnati College of Medicine, UNITED STATES

## Abstract

**Background:**

Understanding the patterns of HCV RNA levels during acute hepatitis C virus (HCV) infection provides insights into immunopathogenesis and is important for vaccine design. This study evaluated patterns of HCV RNA levels and associated factors among individuals with acute infection.

**Methods:**

Data were from an international collaboration of nine prospective cohorts of acute HCV (InC^3^ Study). Participants with well-characterized acute HCV infection (detected within three months post-infection and interval between the peak and subsequent HCV RNA levels≤120 days) were categorised by *a priori*-defined patterns of HCV RNA levels: i) spontaneous clearance, ii) partial viral control with persistence (≥1 log IU/mL decline in HCV RNA levels following peak) and iii) viral plateau with persistence (increase or <1 log IU/mL decline in HCV RNA levels following peak). Factors associated with HCV RNA patterns were assessed using multinomial logistic regression.

**Results:**

Among 643 individuals with acute HCV, 162 with well-characterized acute HCV were identified: spontaneous clearance (32%), partial viral control with persistence (27%), and viral plateau with persistence (41%). HCV RNA levels reached a high viraemic phase within two months following infection, with higher levels in the spontaneous clearance and partial viral control groups, compared to the viral plateau group (median: 6.0, 6.2, 5.3 log IU/mL, respectively; *P*=0.018). In the two groups with persistence, *Interferon lambda 3* (*IFNL3*) CC genotype was independently associated with partial viral control compared to viral plateau (adjusted odds ratio [AOR]: 2.75; 95%CI: 1.08, 7.02). In the two groups with viral control, female sex was independently associated with spontaneous clearance compared to partial viral control (AOR: 2.86; 95%CI: 1.04, 7.83).

**Conclusions:**

Among individuals with acute HCV, a spectrum of HCV RNA patterns is evident. *IFNL3* CC genotype is associated with initial viral control, while female sex is associated with ultimate spontaneous clearance.

## Introduction

Initial infection with hepatitis C virus (HCV) is characterized by detection of virus in blood within 2–14 days of exposure, increases in hepatic transaminases, and appearance of detectable HCV-specific antibodies (anti-HCV) within 30–60 days of exposure [1–6]. Understanding of early HCV RNA patterns during acute HCV infection provides insights into immunopathogenesis and is important for vaccine design. However, data in this area remains limited due to the generally asymptomatic nature of initial infection, the highly marginalised nature of at-risk populations, such as people who inject drugs (PWID), and small study populations.

Based on available data, the dynamics of HCV RNA levels following HCV exposure indicate an initial “pre-ramp-up” phase with intermittent low-level viraemia (from exposure to initial quantifiable HCV RNA), followed by a “ramp-up” phase with an exponential increase in HCV RNA levels (8–10 days), and a high viraemic plateau phase (45–68 days)[2]. Among the ~25% with subsequent spontaneous clearance [7, 8], this plateau phase is followed by a decline in HCV RNA to undetectable levels [9–12]. Among those who subsequently develop persistent HCV infection, patterns of HCV RNA levels are heterogeneous [3, 12–15], but there are limited studies investigated the differential patterns of HCV RNA levels in these individuals. A study of acute HCV infection among HIV co-infected men who have sex with men characterised those who developed persistent infection into two broad patterns: 1) fluctuating levels of HCV RNA (≥1 log IU/mL) corresponding to partial viral control with subsequent persistence; or 2) stable high HCV RNA levels [11, 16].

Factors associated with spontaneous HCV clearance have been well-described and include host (e.g. female sex, *Interferon lambda 3 [IFNL3]* genotype [formerly called *IL28B*], immune responses) [7, 8, 15, 17–21] and viral factors (e.g. HCV genotype and viral evolution) [8, 11, 22–25]. However, little is known about the host and viral factors associated with partial viral control with persistence when compared to either those who achieve spontaneous HCV clearance or those without partial viral control (high viral plateau with persistence).

The International Collaboration of Incident HIV and Hepatitis C in Injecting Cohorts (InC^3^) Study, is a collaborative of pooled data from nine prospective cohorts mainly following PWID [26], consisting of well-characterized participants with acute HCV infection. This current study assessed the dynamics of HCV RNA and alanine aminotransferase (ALT) levels in acute HCV infection among those with spontaneous clearance and persistent infection. Individuals with persistent infection were further categorized into those with high viral plateau with persistence and partial viral control with persistence.

## Materials & Methods

### Study population and design

The InC^3^ Study is a collaboration of nine prospective cohorts evaluating HIV and HCV infection outcomes [26] All cohorts follow participants at regular intervals using standardized methods, although participants were recruited and followed over different time periods, between 1985 and 2010. The InC^3^ Study includes both: 1) participants without HCV infection; and 2) participants with acute HCV infection. All participants provided written informed consent and the cohort protocols conform to the ethical guidelines of the 1975 Declaration of Helsinki as reflected in *a priori* approval by the University of California at San Francisco (UCSF) Institutional Review Board. The cohort protocols were also approved by local ethics committees in each site [26].

For the current study, only individuals with acute HCV were included. Acute HCV was defined as either: 1) HCV seroconversion with an anti-HCV negative test followed by either an anti-HCV or HCV RNA positive test within two years of the anti-HCV negative test; or 2) evidence of symptomatic HCV infection. Symptomatic HCV infection was defined as a) a positive anti-HCV/HCV RNA test; b) jaundice or ALT elevation >400 U/L; and c) detection of HCV RNA or history of high-risk exposure within three months of clinical manifestation of acute HCV.

The estimated date of HCV infection was calculated based on a hierarchy using all serological (anti-HCV), virological (HCV RNA) and clinical (symptoms and liver function tests) data to arrive at the most precise estimate of infection date:

- Among individuals with HCV RNA positive and anti-HCV negative at acute HCV detection, date of infection was four weeks prior to HCV RNA detection [3, 6].
- Among individuals with symptomatic acute HCV, date of infection was six weeks prior to its onset (jaundice or ALT >400 IU/L) [27].
- Among individuals with a negative anti-HCV test followed by either a positive anti-HCV or HCV RNA test, seroconversion was assumed to occur at the mid-point between the last negative and the first positive test. HCV seroconversion generally occurs about 30–60 days following infection [3, 6, 28]. Date of infection in this group was six weeks prior to estimated seroconversion date if the first positive test was anti-HCV test and four weeks prior to estimated seroconversion date if the first positive test was only HCV RNA test.

**Fig 1:**
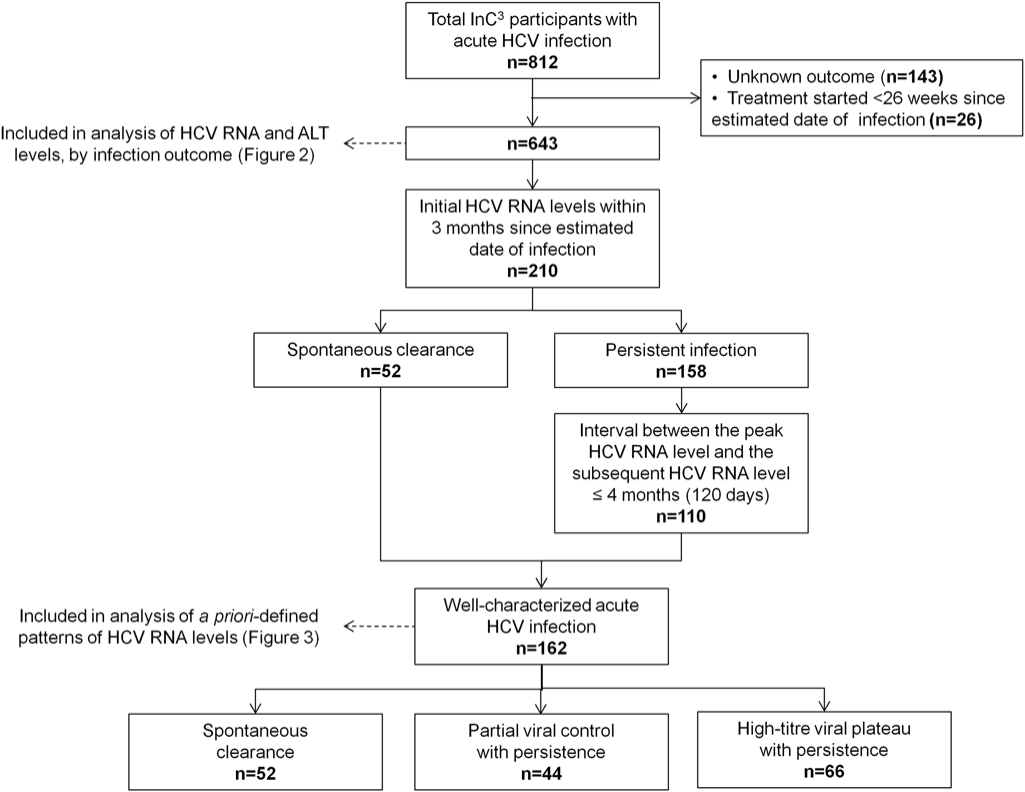
Overview of InC^3^ study population.

Profiles of levels of HCV RNA and ALT in those with spontaneous clearance and persistent infection were assessed in 643 (79%) of the 812 persons with acute HCV infection in the InC^3^ study (Fig. 1). Of the 812, 143 (18%), individuals with unknown virological outcome were excluded. Individuals treated for HCV within 26 weeks of estimated duration of infection were also excluded (n = 37; 5%) to reduce misclassification bias resulting from uncertainty around subsequent spontaneous clearance in the absence of treatment [8, 21].

Among untreated individuals, all longitudinal HCV RNA and ALT measurements were used to assess the profiles of HCV RNA and ALT levels. Among treated individuals (treatment commencement after 26 weeks following infection), only HCV RNA and ALT data up to and including the date of treatment commencement were included in the analysis. Individuals with documented re-infection episodes after spontaneous clearance were censored at the last undetectable HCV RNA before re-infection [29].

Patterns of HCV RNA levels were then evaluated in a sub-group of people classified as “well-characterized acute HCV infection” (Fig. 1). First, the study population was restricted to individuals with HCV RNA detected within the first three months following estimated date of infection (n = 210). This reduced the likelihood that changes in early HCV RNA levels would be missed due to late detection of initial HCV RNA. Two hundred and ten participants met this criteria including 52 with spontaneous clearance and 158 with persistent infection. Second, for participants with persistent infection to be classified as having “well-characterized acute HCV infection” a second HCV RNA test within four months (120 days) of their peak HCV RNA test was also required. This reduced the likelihood of missing unobserved HCV RNA values among individuals with persistent infection and wide intervals between consecutive HCV RNA tests. One hundred and ten participants with persistent infection met this criteria. As such, 162 paticipants were classified as “well-characterized acute HCV infection” (Fig. 1).

### Study outcomes

Spontaneous clearance was defined by two consecutive undetectable HCV RNA test results ≥4 weeks apart following the estimated date of infection [8]. Individuals with persistent infection were categorized into two broad *a priori*-defined groups. Partial viral control with persistence was defined by a ≥1 log IU/mL decline between the peak HCV RNA level during the first 90 days following infection and the subsequent HCV RNA level (within 120 days from peak). This group represented individuals who had an initial ≥1 log IU/mL decline in HCV RNA level and stayed at low levels, or had an initial ≥1 log IU/mL decline in HCV RNA levels and had a re-increase (fluctuation) during follow-up (for representative examples, see S1 Fig. [A, B, C]). High viral plateau with persistence (hereinafter called viral plateau with persistence) was defined by an increase or <1 log IU/mL decline between the peak HCV RNA level during the first 90 days following infection and the subsequent HCV RNA level (within 120 days from peak). This group represented individuals who had an increase in HCV RNA level after the first 90 days following infection, or had relatively stable high HCV RNA levels with <log IU/mL initial decline (for representative examples, see S1 Fig. [D, E, F]). For participants with more than one HCV RNA level within 120 days after the peak level,both individual values and medians were assessed. As results were similar, results with median values are presented.

Among individuals with any of the above described patterns of HCV RNA levels, those with undetectable HCV RNA followed by detectable HCV RNA during twelve-month follow-up were classified as having intermittent viraemia. Among those with intermittent viraemia, viral genotype/subtype data and viral sequence analysis were used to distinguish three subcategories: reinfection (heterologous virus with no subsequent detection of the original viral strain), intercalation (homologous virus), and indeterminate (viral sequencing unavailable, or heterologous virus with subsequent detection of the original viral strain) [29].

### Laboratory testing

Choice of qualitative and quantitative HCV RNA testing varied by cohort but was consistent at each site. Qualitative HCV RNA testing was performed using the following assays: Versant TMA [Bayer, Australia;<10 IU/ml], COBAS AmpliPrep/COBAS TaqMan (Roche, Branchburg, NJ, USA;<15 IU/ml), COBAS AMPLICOR HCV Test v2.0 (Roche Diagnostics, Mannheim, Germany;<50 IU/ml) or discriminatory HCV transcription-mediated amplification component of the Procleix HIV-1/HCV (Gen-Probe, San Diego, CA, USA;<12 copies/mL). Quantitative HCV RNA testing was performed using the Versant HCV RNA 3.0 (Bayer, Australia;<615 IU/ml), COBAS AMPLICOR HCV MONITOR 2.0 (Roche Diagnostics, Mannheim, Germany;<600 IU/ml), COBAS AmpliPrep/COBAS TaqMan (Roche, Branchburg, NJ, USA;<15 IU/ml) or an in-house PCR (<1000 IU/ml) [30, 31]. HCV genotype was determined by line-probe assay (Versant LiPa1/LiPa2, Bayer, Australia) or HCV sequencing at acute HCV detection. Among those with undetectable HCV RNA (no genotype) and available samples, Murex HCV serotyping was performed to determine HCV genotype (Murex Biotech Limited, Dartford, UK). *IFNL3* genotyping was determined by sequencing of the rs12979860 single nucleotide polymorphism, as previously described [8, 18, 19, 21].

### Statistical analyses

Population-average curves describing HCV RNA and ALT profiles were constructed using longitudinal HCV RNA and ALT measurements. The median HCV RNA levels (log IU/mL) and ALT levels (IU/L) among all individuals (n = 643) were calculated in monthly intervals for the first 12 months following estimated date of infection. To calculate monthly medians of HCV RNA and ALT levels, all individual measurements recorded during each month were included. Medians of HCV RNA and ALT levels were compared between groups using Wilcoxon-Mann-Whitney test or Kruskal Wallis test. Similar analyses were performed in those with well-characterized acute HCV infection (n = 162).

To account for within-individual clustering of data points (repeated measurements) and the natural heterogeneity of the population a random effects linear regression model was fitted for the different patterns of HCV RNA levels. Overall time trends were allowed to vary smoothly using natural cubic splines [32]. The intercept and the slope were allowed to differ per individual via the random effects.

Factors associated with patterns of HCV RNA levels were assessed among those with well-characterized acute HCV infection (n = 162). Hypothesized factors were determined *a priori* based on factors known to be associated with spontaneous clearance of HCV infection and included age [33]. Sex [7, 8, 15, 17, 18], ethnicity [34], *IFNL3* genotype (SNP rs12979860; CC vs. CT/TT) [8, 19–21], HIV co-infection [34], and HCV genotype (genotype 1 vs. genotype non-1) [8, 11, 22]. Due to the data of higher spontaneous clearance in HCV genotype 1 [8, 11, 22], and the small numbers within some genotype categories (2/4/6/mixed), all HCV genotype non-1 infections were grouped together.

first, the distribution of each hypothesised factor was compared between those with spontaneous clearance, partial viral control with persistence and viral plateau with persistence using chi square for categorical variables and kruskal wallis test for continuous variables. a multinomial logistic regression model was used for multivariate analyses, including factors with an overall *P* value<0.20 in unadjusted analysesl. two separate models were fitted considering viral plateau with persistence and partial viral control with persistence as the baselines, respectively. given that peak hcv rna levels (iu/ml) and log10 transformation (log iu/ml) of these data was not normally distributed, a dichotomised version of this variable was included in the model using a threshold of 400,000 iu/ml (5.6 log iu/ml) [35]. statistically significant differences were assessed at *P*<0.05; p-values were two-sided. all analyses were performed using stata v12.0 (college station, tx, united states).

## Results

### Participant characteristics

The characteristics of the population with acute HCV infection included in this study (n = 643) are summarized in Table 1. Median age was 26 years, 36% were female, 96% had a history of injecting drug use, and 17% (n = 112) received HCV treatment during follow-up (all treated individuals included started treatment at >26 weeks following infection). Acute HCV infection was documented by HCV seroconversion in 89% (n = 573) of participants; 183 of these were HCV RNA positive and anti-HCV negative at acute HCV detection. During the first 12 months following estimated date of HCV infection, a median of three HCV RNA tests (inter-quartile range [IQR]: 2, 5), with a median of 35 days (IQR: 23, 91) between tests, were included. The median interval from estimated date of infection to the first positive anti-HCV or HCV RNA test was 101 days (IQR: 28, 172). The overall median follow-up time from the estimated date of infection to the last HCV RNA measurement was 19 months (IQR: 9, 36).

**Table 1:** Characteristics of individuals with acute HCV infection in the InC^3^ Study.

	Number (%)*
	*Overall (n = 643)*
**Site**	
ACS (the Netherlands)	44 (7)
ATAHC (Australia)	125 (19)
BAHSTION (United States)	50 (8)
BBAASH (United States)	114 (17)
HEPCO (Canada)	78 (12)
HITS-c (Australia)	10 (2)
HITS-p (Australia)	90 (14)
N2 (Australia)	17 (3)
UFO (United States)	115 (18)
Median age at the time of HCV infection, yrs (IQR**)	26 (23, 33)
**Sex**	
Female	230 (36)
Male	411 (64)
Unknown	2 (<1)
**Ethnicity**	
Caucasian	525 (82)
Black	24 (4)
Indigenous	32 (5)
Other	47 (7)
Unknown	15 (2)
**History of injecting drug use**	616 (96)
**Documented acute HCV infection by:**	
HCV seroconversion	573 (89)
Symptomatic infection, and a recent history of high-risk exposure	70 (11)
**Clinical illness at the time of acute HCV infection**	
No	120 (19)
Yes	139 (22)
Unknown	384 (61)
***IFNL3* genotype (rs12979860)**	
TT	64 (10)
CT	215 (33)
CC	272 (42)
Unknown	92 (14)
**HIV status at the time of HCV infection**	
Negative	574 (89)
Positive	44 (7)
Unknown	25 (4)
**HCV genotype**	
Genotype 1	302 (47)
Genotype 2	33 (5)
Genotype 3	187 (29)
Genotype 4	7 (1)
Genotype 6	4 (1)
Mixed genotype	14 (2)
Unknown	96 (15)

* Percentages indicate column percentages;

** IQR: Inter-quartile range

### HCV RNA and ALT levels during acute infection, by infection outcome

**Fig 2:**
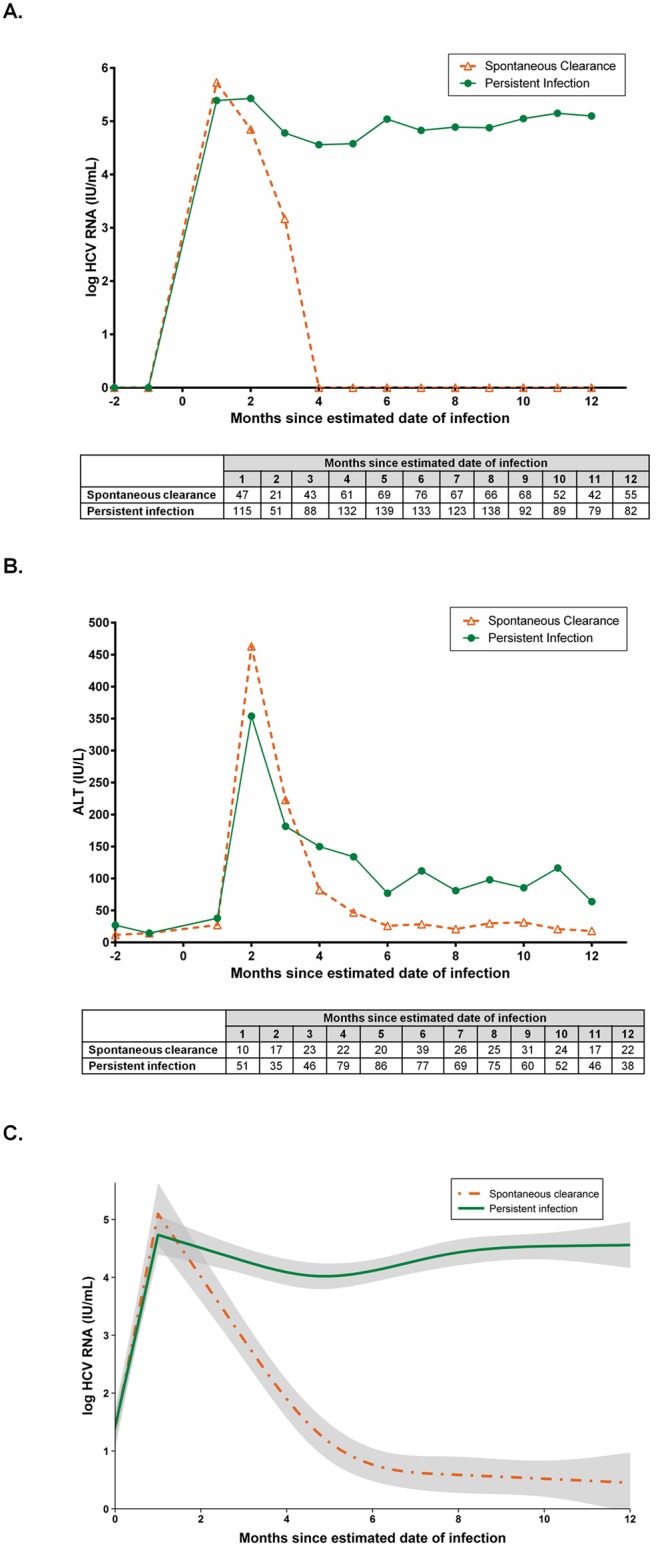
Monthly medians of HCV RNA and ALT levels in individuals with acute HCV infection in the InC^3^ study (total n = 643). (A) HCV RNA levels, by infection outcome (clearance vs. persistence); (B) ALT levels by infection outcome (clearance vs. persistence), tables underneath panel A and B represent number of participants with available HCV RNA/ALT levels measurements at each time point; (C) Fitted HCV RNA patterns, shaded areas in panel C represent the 95% confidence intervals.

HCV RNA and ALT levels among those with spontaneous clearance and persistent infection were assessed among 643 participants with acute HCV infection (173 with spontaneous clearance and 470 with persistent infection; Fig. 2).

Peak median HCV RNA levels occurred at one month following infection in those with spontaneous clearance (5.7 log IU/mL; IQR: 3.0, 7.0) and at months one and two in those with persistent infection (month one: 5.4 log IU/mL; IQR: 3.8, 6.5 and month two: 5.4 log IU/m; IQR: 3.1, 6.4; *P* = 0.39 for month one comparison). By month two to four, HCV RNA declines were seen in both groups. At month two, median HCV RNA levels remained comparable between individuals with persistent infection (5.4 log IU/mL; IQR: 3.1, 6.4) and spontaneous clearance (4.8 log/IU/mL; IQR: 0.0, 6.0; *P* = 0.38). Median HCV RNA levels initially diverged at three months following infection, being 4.8 log/IU/mL (IQR: 3.3, 6.0) in individuals with persistent infection compared to 3.2 log/IU/mL (IQR: 0.0, 6.1) in those with spontaneous clearance (*P* = 0.03). Subsequent marked divergence in median HCV RNA level between the two groups was seen at month four and beyond (Fig. 2A).

Peak HCV RNA preceded peak ALT by approximately one month, with peak ALT levels being observed in month two following infection among individuals with persistent infection (354 IU/L; IQR: 99, 944) and spontaneous clearance (463 IU/L; IQR: 72, 2316; *P* = 0.43). By month four to five following infection, median ALT level had declined sharply in both groups. Among individuals with spontaneous clearance, median ALT returned to normal at month five and remained within the normal range thereafter. In contrast, in those with persistent infection, median ALT levels remained elevated throughout follow-up with intermittent fluctuations (Fig. 2B). Similar results were observed in sensitivity analyses (S2 Fig.) restricted to individuals identified in the so called sero-silent acute HCV phase (HCV RNA positive and anti-HCV negative) given the well-defined estimated date of infection in this sub-group (n = 183).

### Patterns of HCV RNA levels among individuals with well-characterized acute HCV infection

To further characterise the different patterns of HCV RNA levels, longitudinal HCV RNA levels were assessed among individuals with well-characterized acute HCV infection (n = 162). Compared to those not included in this analysis (n = 481), included individuals were younger, were less likely to be Caucasians, and more likely to have sympotomatic acute infection (S1 Table).

**Fig 3:**
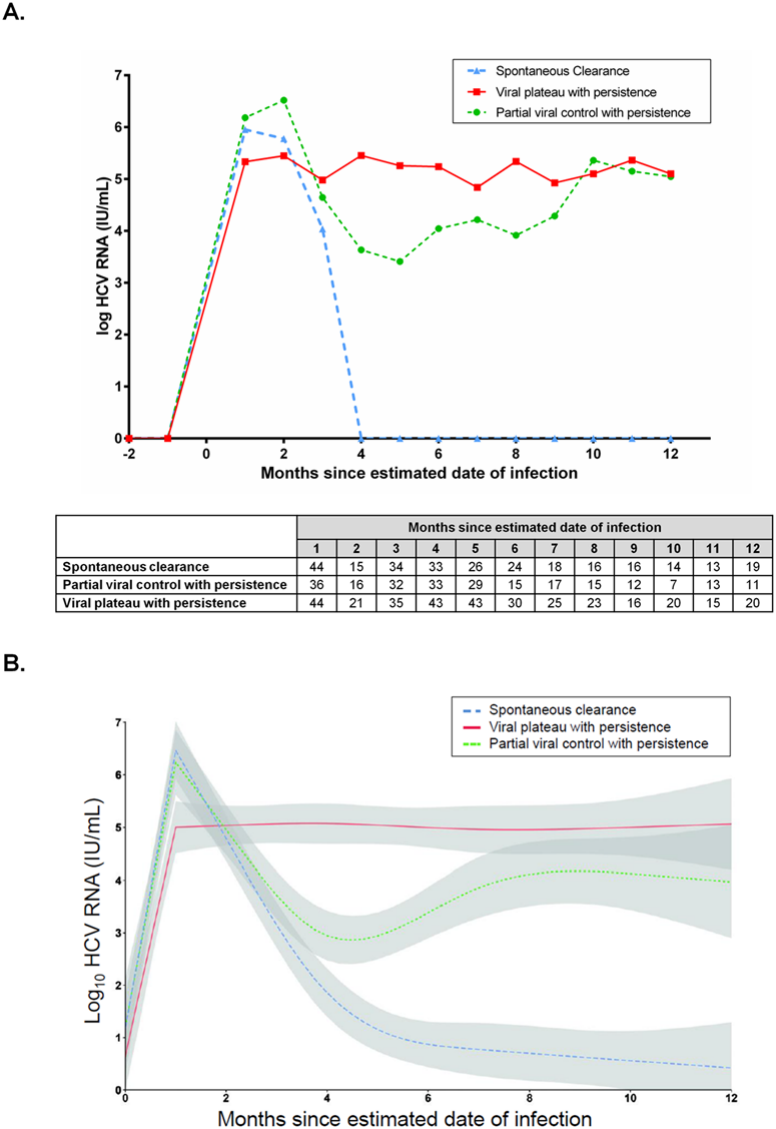
Patterns of HCV RNA levels in individuals with well-characterized acute HCV infection in the InC^3^ study (total n = 162). (A) Monthly medians of HCV RNA levels, table underneath represents number of participants with available HCV RNA level measurements at each time point; (B) Fitted HCV RNA patterns, shaded areas represent the 95% confidence intervals.

Individuals with well-characterized acute HCV infection (n = 162) had a median of 4.5 HCV RNA tests (IQR: 3, 8), with a median of 33 days (IQR: 27, 68) between tests during the first 12 months following estimated date of HCV infection. Median interval from estimated date of infection to the first positive anti-HCV or HCV RNA test was 28 days (IQR: 28, 49). Spontaneous clearance was observed in 52 individuals. Among those with persistent infection (n = 110), 44 individuals demonstrated partial viral control with persistence (defined by ≥1 log IU/mL decline between the peak and the subsequent HCV RNA levels) and 66 individuals demonstrated viral plateau with persistence (defined by increase or <1 log IU/mL decline between the peak and the subsequent HCV RNA levels). HCV RNA levels among individuals with these three HCV RNA patterns are illustrated in Fig. 3.

Among 66 individuals with viral plateau and persistence, consistently high HCV RNA levels with irregular fluctuations were observed throughout follow-up. Representative examples of HCV RNA levels among individual cases are shown in S1 Fig. (D, E, F). Intermittent viraemia was observed in four participants in this group during twelve-months of follow-up, including one case of intercalation, three indeterminate cases, and no re-infections.

Among 44 individuals with partial viral control and persistence, HCV RNA levels declined after the peak and rebounded following months 4–5. Representative examples of HCV RNA levels among individual cases are shown in S1 Figs. (A, B, C). The decline in median HCV RNA levels between month two and five was 3.7 log IU/mL. HCV RNA levels observed after viral rebound were lower than the initial values. The patterns of HCV RNA levels were relatively similar between individuals with partial control and those with viral plateau after months 8–10 following infection. Intermittent viraemia was observed in 10 participants with partial control and persistence during twelve months of follow-up, including six cases of intercalation, four indeterminate cases, and no re-infections.

Median (IQR) HCV RNA levels at month one following infection were 6.0 log IU/mL (4.3, 7.1), 6.2 log IU/mL (5.2, 7.1), and 5.3 log IU/mL (4.1, 6.0) in individuals with spontaneous clearance, partial viral control with persistence, and viral plateau with persistence, respectively (*P* = 0.02). Compared to those with viral plateau with persistence, median HCV RNA levels was significantly higher in participants with spontaneous clearance (*P* = 0.03) and partial viral control with persistence (*P*<0.01). However, median HCV RNA levels did not significantly differ between participants with partial viral control compared to those with clearance (*P* = 0.68).

### Factors associated with patterns of HCV RNA levels

**Fig 4:**
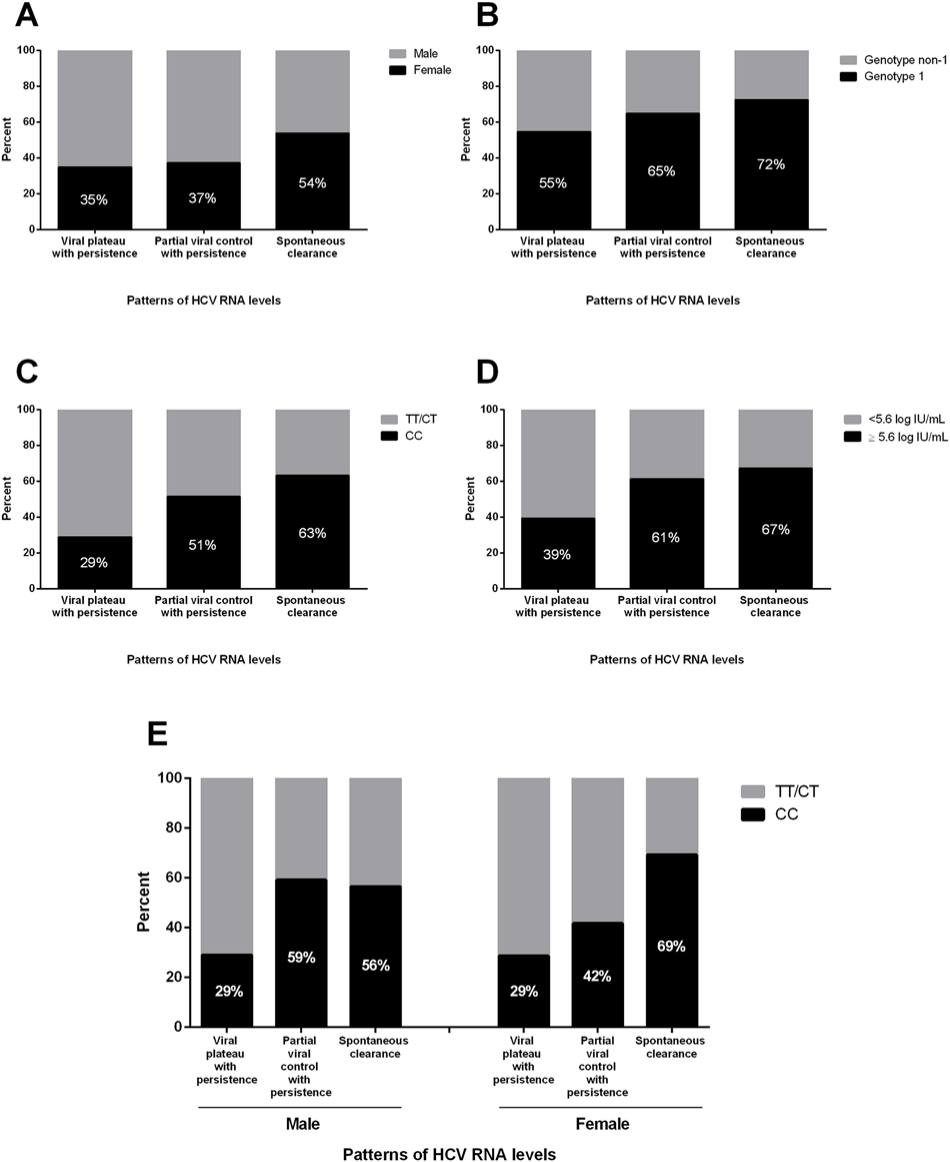
Distribution of sex, HCV genotype, *IFNL3* genotype and peak HCV RNA levels by patterns of HCV RNA levels in individuals with acute HCV infection in the InC^3^ study. (A) Sex, *P* = 0.09; (B) HCV genotype, *P* = 0.18; (C) *IFNL3* genotype, *P*<0.01; (D) Peak HCV RNA levels, *P*<0.01; (E) *IFNL3* genotype stratified by sex, male *P* = 0.03, female *P* = 0.02.

Among individuals with well-characterized acute HCV, key baseline characteristics were compared among those with viral plateau with persistence (n = 66), partial viral control with persistence (n = 44) and spontaneous clearance (n = 52; Table 2, Fig. 4). Between the three groups, there was a significant difference in the proportion with *IFNL3* CC genotype (29% vs. 51% vs. 63%, respectively; *P*<0.01), median peak HCV RNA level (5.3 log IU/mL vs. 6.0 log IU/mL vs. 6.5 log IU/mL, respectively; *P*<0.01) and the proportion with peak HCV RNA ≥5.6 log IU/mL (39% vs. 61% vs. 67%, respectively; *P*<0.01). There was also a trend towards a significant difference in sex across the three groups, with 54% of individuals with spontaneous HCV clearance being female, as compared to 37% in those with partial viral control with persistence and 35% in those with viral plateau with persistence (*P* = 0.09).

**Table 2:** Distribution of selected demographic, clinical and virologic variables by various patterns of HCV RNA levels in individuals with well-characterized acute HCV infection in the InC^3^ study (n = 162).

	Viral plateau with persistence (n = 66) n (%)*	Partial viral control with persistence (n = 44) n (%)*	Spontaneous clearance (n = 52) n (%)*	*P (across three groups)*	*P (Viral plateau vs*. *Partial viral control)*
Median age, yrs (IQR**)	24 (21, 28)	24 (22, 30)	24 (21, 28)	0.66	0.54
**Sex**				0.09	0.80
Male	43 (65)	27 (63)	24 (46)		
Female	23 (35)	16 (37)	28 (54)		
**Ethnicity**				0.65	0.89
Caucasian	49 (78)	35 (81)	44 (85)		
Black	7 (11)	3 (7)	1 (2)		
Indigenous	2 (3)	1 (2)	1 (2)		
Other	5 (8)	4 (9)	6 (11)		
***IFNL3* genotype (rs12979860)**				<0.01	0.03
TT/CT	42 (71)	17 (49)	18 (37)		
CC	17 (29)	18 (51)	31 (63)		
**HIV status**				0.68	0.65
Negative	61 (95)	42 (98)	48 (98)		
Positive	3 (5)	1 (2)	1 (2)		
**HCV genotype**				0.18	0.32
Genotype non-1	29 (45)	13 (35)	11 (27)		
Genotype 1	35 (55)	24 (65)	29 (72)		
Median peak HCV RNA levels during three months following infection, log IU/mL (IQR**)	5.3 (3.6–6.0)	6.0 (5.1–7.2)	6.5 (5.3–7.2)	<0.01	<0.01
**Peak HCV RNA levels during three months following infection, log IU/mL**				<0.01	0.02
<5.6 log IU/mL	40 (61)	17 (39)	17 (33)		
≥5.6 log IU/mL	26 (39)	27 (61)	35 (67)		
**Presentation of acute HCV infection**				0.35	0.24
Simultaneous HCV RNA+ and anti-HCV-	40 (61)	33 (75)	37 (71)		
Asymptomatic seroconversion^†^	18 (27)	9 (20)	12 (23)		
Symptomatic acute HCV^‡^	8 (12)	2 (5)	3 (6)		

* Percentages indicate column percentages

** IQR: Inter-quartile range

^†^ A negative anti-HCV test followed by a positive anti-HCV/HCV RNA test

^‡^ A positive anti-HCV/HCV RNA test and jaundice or ALT elevation >400 U/L

As female sex may have a role in modifying the association of *IFNL3* genotype with HCV viral control [8, 18], the proportion of *IFNL3* CC genotype was assessed stratified by sex. As shown in Fig. 4E, the difference in the proportion of *IFNL3* CC genotype was more pronounced among females compared to males when comparing those with partial viral control with persistence and spontaneous clearance.

Table 3 presents multinomial logistic regression models of factors associated with viral plateau with persistence, partial viral control with persistence and spontaneous clearance. *IFNL3* genotype, sex, HCV genotype and peak HCV RNA levels, were included in subsequent models, given the associations observed in unadjusted analyses (*P*<0.20). In the two groups with viral persistence, the only factor independently associated with partial viral control (compared to viral plateau) was *IFNL3* CC genotype (adjusted odds ratio [AOR]: 2.75; 95% CI: 1.08, 7.02; *P* = 0.03). In the two groups with viral control, the only factor independently associated with spontaneous clearance (compared to partial viral control with persistence) was female sex (AOR: 2.86; 95% CI: 1.04, 7.83; *P* = 0.04). Lastly, female sex (AOR: 3.10; 95% CI: 1.18, 8.17; *P* = 0.02), *IFNL3* CC genotype (AOR: 5.00; 95% CI: 1.85, 13.51; *P*<0.01), HCV genotype 1 (AOR: 3.50; 95% CI: 1.24, 9.87; *P* = 0.02), and peak HCV RNA level≥5.6 log IU/mL (AOR: 3.77; 95% CI: 1.38, 10.28; *P* = 0.01) were all independently associated with spontaneous clearance compared to viral plateau with persistence.

**Table 3:** Adjusted multinomial logistic regression model assessing factors associated with patterns of HCV RNA levels in individuals with well-characterized acute HCV infection in the InC^3^ study*.

	Partial viral control with persistence vs. Viral plateau with persistence** AOR^†^ (95% CI)	*P*	Spontaneous clearance vs. Partial viral control with persistence** AOR^†^ (95% CI)	*P*	Spontaneous clearance vs. Viral plateau with persistence** AOR^†^ (95% CI)	*P*
**Sex**						
Male	1.00		1.00		1.00	
Female	1.08 (0.42, 2.82)	0.87	2.86 (1.04, 7.83)	0.04	3.10 (1.18, 8.17)	0.02
***IFNL3* genotype (rs12979860)**						
TT/CT	1.00		1.00		1.00	
CC	2.75 (1.08, 7.02)	0.03	1.82 (0.64, 5.12)	0.26	5.00 (1.85, 13.51)	<0.01
**HCV genotype**						
Genotype non-1	1.00		1.00		1.00	
Genotype 1	1.65 (0.65, 4.20)	0.29	2.11 (0.72, 6.21)	0.17	3.50 (1.24, 9.87)	0.02
**Peak HCV RNA levels**						
<5.6 log IU/mL	1.00		1.00		1.00	
≥5.6 log IU/mL	2.15 (0.85, 5.41)	0.10	1.75 (0.59, 5.18)	0.31	3.77 (1.38, 10.28)	0.01

*Included 128 participants in the model

**Baseline comparison group

^†^ AOR: Adjusted Odds Ratio

## Discussion

This study has characterized the patterns of HCV RNA and ALT levels in a large population with well-defined acute HCV infection, the majority of whom were PWID. Following acute HCV infection, HCV RNA levels reached a high viraemic phase followed by either spontaneous clearance or persistent infection, with the divergence of HCV RNA levels occurring at approximately three months following infection. Two broad patterns of HCV RNA levels were designated *a priori* among individuals with persistent infection in this study, including viral plateau with persistence and partial viral control with persistence. *IFNL3* CC genotype and female sex emerged as the most predictive factors for viraemia patterns. Among individuals who develop viral persistence (viral plateau and partial viral control), *IFNL3* CC genotype appears to be the most predictive factor of initial viral control. Furthermore, among those who exhibit some degree of viral control (clearance or partial viral control with persistence), female sex is particularly important for determining whether someone will ultimately spontaneously clear the infection.

Differential patterns of HCV RNA levels may reflect a spectrum of immunological viral control during acute infection. Partial viral control with persistence is indicative of a complex pattern of virus–host interaction and can represent loss of immunological control of viral replication due to virological escape from initial innate responses or a failure in the kinetics, magnitude or breadth of HCV-specific adaptive immune responses (reviewed in [16, 36]). Rapid viral evolution occurs during acute HCV infection, with a higher genetic diversity observed among those with ultimate persistence as compared to clearance [11, 25]. Reductions in HCV RNA levels and genetic diversity have been identified within 100 days of infection (a “bottleneck” effect) irrespective of infection outcome, with subsequent increased HCV RNA levels and diversity in those with persistent infection [37]. The pattern of HCV RNA levels in those with partial viral control and persistence obsereved in the current study is consistent with these data, given this control was observed largely between months 3–5 (90–150 days). Vigorous but late onset HCV-specific T-cell responses have been also identified in individuals with transient viral control in acute HCV infection, while ultimately loss of HCV-specific T-cell responses has led to recurrent HCV viraemia and persistent infection (reviewed in [36, 38]). Although two broad patterns of HCV RNA levels were defined among individuals with persistent infection in this study, heterogeneity in individual HCV RNA patterns is still evident in these two groups [3, 12–15].

Among individuals with well-characterized acute HCV infection who eventually developed viral persistence, a notable proportion (40%) initially experienced at least one log IU/mL decline in their HCV RNA levels. This finding has clinical implications suggesting that spontaneous clearance cannot be predicted solely based on initial decline in HCV RNA levels.

Among individuals with persistent infection, *IFNL3* CC genotype was independently associated with partial viral control (either a decrease or fluctuation in HCV RNA). Previous studies have demonstrated that genetic variation in the *IFNL3* gene region is associated with both spontaneous and treatment-induced HCV clearance (reviewed in [4, 23]). Our findings suggest that even among individuals who develop persistent infection, *IFNL3* CC genotype plays a role in initial viral control. Some other studies also demonstrated an association between *IFNL3* CC genotype and higher HCV RNA levels in acute infection, regardless of acute HCV outcome [10, 39], and also in chronic infection [40–42]. Although the mechanisms and role of *IFNL3* genotype in viral control and outcome of HCV infection are not fully understood, there is evidence that *IFNL3* regulates the interferon stimulated genes (ISGs), required for initial control of viral infection (reviewed in[43]). Another study demonstrated a superior innate immune function, particularly of natural killer cells, to interferon-based therapy in individuals with *IFNL3* CC genotype, resulting in improved treatment induced viral control [44]. More evidence is needed to show if there is the same association between *IFNL3* genotype and natural killer cells function in acute HCV infection although the existing evidence supports the essential role of natural killer cells in viral control in acute HCV through inducing T-cell responses (reviewed in [45]).

Among individuals with viral control, female sex was independently associated with spontaneous clearance, consistent with the increasing literature demonstrating that females exhibit more successful responses to HCV [7, 8, 15, 17, 18]. However, it is interesting that among those with any level of viral control (partial control or clearance), female sex is particularly important for determining HCV clearance outcome. Unfortunately, data on sex-based differences in immunological profiles in individuals with HCV infection are sparse. However, there is evidence of a lower rate of HCV clearance in men compared to women [7, 8, 15, 17, 18] and also in postmenopausal women compared to premenopausal women [46, 47] implicating female hormones, such as estrogens and progesterone, in viral control.

Female sex, *IFNL3* CC genotype, HCV genotype 1, and higher peak HCV RNA levels were significantly associated with spontaneous clearance when compared to participants with viral plateau and persistence. These findings are consistent with previous data demonstrating the association of female sex [7, 8, 15, 17, 18], *IFNL3* CC genotype [8, 19–21], HCV genotype 1 [8, 11, 22] and peak HCV RNA levels [10] with spontaneous HCV clearance following acute infection.

The current study did not show any significant association between age and the patterns of HCV RNA levels, although previous studies identified higher spontaneous clearance rate in younger individuals [33]. InC^3^ participants are generally young, given HCV transmission among PWID generally occurs in young age. However, this study may not able to explore the potential association of age with HCV RNA patterns due to a narrow age range.

While the current study is unique given the large sample size and well-defined nature of acute HCV infection, there are several limitations. Participating cohorts bring a range of data types and structures presenting issues surrounding both inconsistent measurement and biological data testing protocols (e.g. HCV RNA assays differed across cohorts with different sensitivity, specificity and lower limit of detection). It is important to note that the definition of partial viral control in this study included either a decrease or fluctuation in HCV RNA, so the HCV RNA curves for partial controllers represent the average curve. There was also heterogeneity in HCV RNA and ALT monitoring schedules across cohorts. We were not able to explore the potential association of individual genotypes with HCV RNA patterns, due to the relatively small numbers of some HCV genotypes. Individuals with well-characterised acute HCV infection were not representative of entire InC^3^ participants given significant difference in distribution of some background characteristics between this group and the rest of InC^3^ participants (e.g. age, ethnicity, and sympotomatic acute infection).

In conclusion, this study identified factors associated with three broad patterns of HCV RNA levels during acute HCV infection. These findings have important implications for understanding the immunological and genetic features important for the control of HCV infection, and have implications for HCV vaccine research. Further research is required to better understand the mechanisms behind the association of *IFNL3* genotype on early viral control and the mechanisms explaining the sex-based immune response to HCV infection.

## Supporting Information

S1 Fig Longitudinal HCV RNA levels in six representative individuals with two *a priori*-defined patterns of persistent infection in the InC^3^ study. (A, B, C) Partial viral control with persistence; (D, E, F) Viral plateau with persistence.
(TIF)

S2 Fig Monthly medians of HCV RNA and ALT levels, by infection outcome (clearance vs. persistence), in individuals with HCV RNA positive and anti-HCV negative at the time of acute HCV detection in the InC^3^ study. (A) HCV RNA levels; (B) ALT levels.
(TIF)

S1 Table Characteristics of individuals with acute HCV infection who were included (well-characterized acute HCV) and were not included in analysis of patterns of HCV RNA levels during acute infection. (DOC)

## References

[1] Busch MP. Insights into the epidemiology, natural history and pathogenesis of hepatitis C virus infection from studies of infected donors and blood product recipients. Transfus Clin Biol. 2001;8(3):200–6. 11499958 10.1016/s1246-7820(01)00125-2

[2] Glynn SA, Wright DJ, Kleinman SH, Hirschkorn D, Tu Y, Heldebrant C, et al Dynamics of viremia in early hepatitis C virus infection. Transfusion. 2005;45(6):994–1002. 10.1111/j.1537-2995.2005.04390.x 15934999

[3] Cox AL, Netski DM, Mosbruger T, Sherman SG, Strathdee S, Ompad D, et al Prospective Evaluation of Community-Acquired Acute-Phase Hepatitis C Virus Infection. Clinical Infectious Diseases. 2005;40(7):951–8. 10.1086/428578 15824985

[4] Hajarizadeh B, Grebely J, Dore GJ. Epidemiology and natural history of HCV infection. Nature Review Gastroenterology Hepatology. 2013;10(9):553–62. 10.1038/nrgastro.2013.107 23817321

[5] Grebely J, Matthews GV, Dore GJ. Treatment of acute HCV infection. Nature Review Gastroenterology Hepatology. 2011;8(5):265–74. 10.1038/nrgastro.2011.32 21423258

[6] Page-Shafer K, Pappalardo BL, Tobler LH, Phelps BH, Edlin BR, Moss AR, et al Testing strategy to identify cases of acute hepatitis C virus (HCV) infection and to project HCV incidence rates. Journal of Clinical Microbiology. 2008;46(2):499–506. 18032621 10.1128/JCM.01229-07 PMC2238141

[7] Micallef JM, Kaldor JM, Dore GJ. Spontaneous viral clearance following acute hepatitis C infection: A systematic review of longitudinal studies. Journal of Viral Hepatitis. 2006;13(1):34–41. 16364080 10.1111/j.1365-2893.2005.00651.x

[8] Grebely J, Page K, Sacks-Davis R, van der Loeff MS, Rice TM, Bruneau J, et al The effects of female sex, viral genotype, and IL28B genotype on spontaneous clearance of acute hepatitis C virus infection. Hepatology. 2014;59(1):109–20. 10.1002/hep.26639 23908124 PMC3972017

[9] Villano SA, Vlahov D, Nelson KE, Cohn S, Thomas DL. Persistence of viremia and the importance of long-term follow-up after acute hepatitis C infection. Hepatology. 1999;29(3):908–14. 10.1002/hep.510290311 10051497

[10] Liu L, Fisher BE, Thomas DL, Cox AL, Ray SC. Spontaneous clearance of primary acute hepatitis C virus infection correlated with high initial viral RNA level and rapid HVR1 evolution. Hepatology. 2012;55(6):1684–91. 10.1002/hep.25575 22234804 PMC3330174

[11] Thomson EC, Fleming VM, Main J, Klenerman P, Weber J, Eliahoo J, et al Predicting spontaneous clearance of acute hepatitis C virus in a large cohort of HIV-1-infected men. Gut. 2011;60(6):837–45. 10.1136/gut.2010.217166 21139063 PMC3095479

[12] Hajarizadeh B, Grebely J, Applegate T, Matthews GV, Amin J, Petoumenos K, et al Dynamics of HCV RNA levels during acute hepatitis C virus infection. Journal of Medical Virology. 2014;86(10):1722–9. 10.1002/jmv.24010 25042465 PMC4276420

[13] McGovern BH, Birch CE, Bowen MJ, Reyor LL, Nagami EH, Chung RT, et al Improving the Diagnosis of Acute Hepatitis C Virus Infection with Expanded Viral Load Criteria. Clinical Infectious Diseases. 2009;49(7):1051–60. 10.1086/605561 19725787 PMC2741541

[14] Smith JA, Aberle JH, Fleming VM, Ferenci P, Thomson EC, Karayiannis P, et al Dynamic Coinfection with Multiple Viral Subtypes in Acute Hepatitis C. Journal of Infectious Diseases. 2010;202(12):1770–9. 10.1086/657317 21067369 PMC3107554

[15] Wang CC, Krantz E, Klarquist J, Krows M, McBride L, Scott EP, et al Acute hepatitis C in a contemporary US cohort: modes of acquisition and factors influencing viral clearance. Journal of Infectious Diseases. 2007;196(10):1474–82. 18008226 10.1086/522608

[16] Thomson EC, Smith JA, Klenerman P. The natural history of early hepatitis C virus evolution; lessons from a global outbreak in human immunodeficiency virus-1-infected individuals. Journal of General Virology. 2011;92(10):2227–36. 10.1099/vir.0.033910-0 21775583 PMC3347798

[17] Page K, Hahn JA, Evans J, Shiboski S, Lum P, Delwart E, et al Acute Hepatitis C Virus Infection in Young Adult Injection Drug Users: A Prospective Study of Incident Infection, Resolution, and Reinfection. Journal of Infectious Diseases. 2009;200(8):1216–26. 10.1086/605947 19764883 PMC2821203

[18] van den Berg CHBS, Grady BPX, Schinkel J, van de Laar T, Molenkamp R, van Houdt R, et al Female sex and IL28b, a synergism for spontaneous viral clearance in hepatitis c virus (HCV) seroconverters from a community-based cohort. PLoS One. 2011;6(11):e27555 10.1371/journal.pone.0027555 22110669 PMC3216978

[19] Thomas DL, Thio CL, Martin MP, Qi Y, Ge D, O/'hUigin C, et al Genetic variation in IL28B and spontaneous clearance of hepatitis C virus. Nature. 2009;461(7265):798–801. 10.1038/nature08463 19759533 PMC3172006

[20] Tillmann HL, Thompson AJ, Patel K, Wiese M, Tenckhoff H, Nischalke HD, et al A Polymorphism Near IL28B Is Associated With Spontaneous Clearance of Acute Hepatitis C Virus and Jaundice. Gastroenterology. 2010;139(5):1586–92. 10.1053/j.gastro.2010.07.005 20637200

[21] Grebely J, Petoumenos K, Hellard M, Matthews GV, Suppiah V, Applegate T, et al Potential role for interleukin-28B genotype in treatment decision-making in recent hepatitis C virus infection. Hepatology (Baltimore, Md). 2010;52(4):1216–24. 10.1002/hep.23850 20803561 PMC2947598

[22] Harris HE, Eldridge KP, Harbour S, Alexander G, Teo CG, Ramsay ME. Does the clinical outcome of hepatitis C infection vary with the infecting hepatitis C virus type? J Viral Hepat. 2007;14(3):213–20. 17305887 10.1111/j.1365-2893.2006.00795.x

[23] Grebely J, Prins M, Hellard M, Cox AL, Osburn WO, Lauer G, et al Hepatitis C virus clearance, reinfection, and persistence, with insights from studies of injecting drug users: towards a vaccine. The Lancet Infectious Diseases. 2012;12(5):408–14. 10.1016/S1473-3099(12)70010-5 22541630 PMC3608418

[24] Ray SC, Wang YM, Laeyendecker O, Ticehurst JR, Villano SA, Thomas DL. Acute hepatitis C virus structural gene sequences as predictors of persistent viremia: hypervariable region 1 as a decoy. J Virol. 1999;73(4):2938–46. 10074143 10.1128/jvi.73.4.2938-2946.1999 PMC104053

[25] Farci P, Shimoda A, Coiana A, Diaz G, Peddis G, Melpolder JC, et al The Outcome of Acute Hepatitis C Predicted by the Evolution of the Viral Quasispecies. Science. 2000;288(5464):339–44. 10.1126/science.288.5464.339 10764648

[26] Grebely J, Morris MD, Rice TM, Bruneau J, Cox AL, Kim AY, et al Cohort Profile: The International Collaboration of Incident HIV and Hepatitis C in Injecting Cohorts (InC3) Study. International Journal of Epidemiology. 2012;42(6):1649–59. 10.1093/ije/dys167 23203695 PMC3887561

[27] Hofer H, Watkins-Riedel T, Janata O, Penner E, Holzmann H, Steindl-Munda P, et al Spontaneous viral clearance in patients with acute hepatitis C can be predicted by repeated measurements of serum viral load. Hepatology. 2003;37(1):60–4. 10.1053/jhep.2003.50019 12500189

[28] Busch MP, Page Shafer KA. Acute-phase hepatitis C virus infection: Implications for research, diagnosis, and treatment. Clinical Infectious Diseases. 2005;40(7):959–61. 15824986 10.1086/428583

[29] Sacks-Davis R, Grebely J, Dore GJ, Osburn W, Cox AL, Rice TM, et al. Hepatitis C virus reinfection and spontaneous clearance of reinfection—the InC3 study. The 20th International Symposium on Hepatitis C Virus and Related Viruses; Melbourne, Australia 6–10 October, 2013.

[30] Badr G, Bédard N, Abdel-Hakeem MS, Trautmann L, Willems B, Villeneuve J-P, et al Early Interferon Therapy for Hepatitis C Virus Infection Rescues Polyfunctional, Long-Lived CD8+ Memory T Cells. Journal of Virology. 2008;82(20):10017–31. 10.1128/jvi.01083-08 18667516 PMC2566289

[31] van de Laar TJW, Molenkamp R, van den Berg C, Schinkel J, Beld MGHM, Prins M, et al Frequent HCV reinfection and superinfection in a cohort of injecting drug users in Amsterdam. Journal of Hepatology. 2009;51(4):667–74. 10.1016/j.jhep.2009.05.027 19646773

[32] Durrleman S, Simon R. Flexible regression models with cubic splines. Statistics in Medicine. 1989;8(5):551–61. 10.1002/sim.4780080504 2657958

[33] Zhang M, Rosenberg PS, Brown DL, Preiss L, Konkle BA, Eyster ME, et al Correlates of spontaneous clearance of hepatitis C virus among people with hemophilia. Blood. 2006;107(3):892–7. 10.1182/blood-2005-07-2781 16204310 PMC1895891

[34] Thomas DL, Astemborski J, Rai RM, Anania FA, Schaeffer M, Galai N, et al The natural history of Hepatitis C virus infection: Host, viral, and environmental factors. Journal of the American Medical Association. 2000;284(4):450–6. 10904508 10.1001/jama.284.4.450

[35] Zeuzem S, Rodríguez-Torres M, Rajender Reddy K, Marcellin P, Diago M, Craxi A, et al Optimized threshold for serum HCV RNA to predict treatment outcomes in hepatitis C patients receiving peginterferon alfa-2a/ribavirin. Journal of Viral Hepatitis. 2012;19(11):766–74. 10.1111/j.1365-2893.2012.01624.x 23043383

[36] Bowen DG, Walker CM. Adaptive immune responses in acute and chronic hepatitis C virus infection. Nature. 2005;436(7053):946–52. 16107834 10.1038/nature04079

[37] Bull RA, Luciani F, McElroy K, Gaudieri S, Pham ST, Chopra A, et al Sequential Bottlenecks Drive Viral Evolution in Early Acute Hepatitis C Virus Infection. PLoS Pathog. 2011;7(9):e1002243 10.1371/journal.ppat.1002243 21912520 PMC3164670

[38] Heller T, Rehermann B. Acute hepatitis C: A multifaceted disease. Seminars in Liver Disease. 2005;25(1):7–17. 15731994 10.1055/s-2005-864778

[39] Hajarizadeh B, Grady B, Page K, Kim AY, McGovern BH, Cox AL, et al Interferon lambda 3 genotype predicts hepatitis C virus RNA levels in early acute infection among people who inject drugs: The InC3 Study. Journal of Clinical Virology. 2014;61(3):430–4. 10.1016/j.jcv.2014.08.027 25256151 PMC4279031

[40] Grady BP, Prins M, Rebers S, Molenkamp R, Geskus RB, Schinkel J. BMI, male sex and IL28B genotype associated with persistently high hepatitis C virus RNA levels among chronically infected drug users up to 23 years following seroconversion. J Viral Hepat. 2015;22(3):263–71. 10.1111/jvh.12303 25174990

[41] Hajarizadeh B, Grady B, Page K, Kim AY, McGovern BH, Cox AL, et al Factors associated with hepatitis C virus RNA levels in early chronic infection: the InC3 study. Journal of Viral Hepatitis. 2015 10.1111/jvh.12384. PMC4496327 25580520

[42] Uccellini L, Tseng FC, Monaco A, Shebl FM, Pfeiffer R, Dotrang M, et al HCV RNA levels in a multiethnic cohort of injection drug users: human genetic, viral and demographic associations. Hepatology. 2012;56(1):86–94. 10.1002/hep.25652 22331649 PMC3369001

[43] Balagopal A, Thomas DL, Thio CL. IL28B and the Control of Hepatitis C Virus Infection. Gastroenterology. 2010;139(6):1865–76. 10.1053/j.gastro.2010.10.004 20950615 PMC3072961

[44] Naggie S, Osinusi A, Katsounas A, Lempicki R, Herrmann E, Thompson AJ, et al Dysregulation of innate immunity in hepatitis C virus genotype 1 IL28B-unfavorable genotype patients: Impaired viral kinetics and therapeutic response. Hepatology. 2012;56(2):444–54. 10.1002/hep.25647 22331604 PMC3361636

[45] Ahlenstiel G. The Natural Killer Cell Response to HCV Infection. Immune Netw. 2013;13(5):168–76. 10.4110/in.2013.13.5.168 24198741 PMC3817297

[46] Floreani A, Cazzagon N, Boemo DG, Baldovin T, Baldo V, Egoue J, et al Female patients in fertile age with chronic hepatitis C, easy genotype, and persistently normal transaminases have a 100% chance to reach a sustained virological response. European Journal of Gastroenterology and Hepatology. 2011;23(11):997–1003. 10.1097/MEG.0b013e32834ae863 21915057

[47] Villa E, Karampatou A, Camm C, Di Leo A, Luongo M, Ferrari A, et al Early menopause is associated with lack of response to antiviral therapy in women with chronic hepatitis C. Gastroenterology. 2011;140(3):818–29. 10.1053/j.gastro.2010.12.027 21167831

