# Supplementary material for: Patterns of Hepatitis C Virus RNA Levels during Acute Infection: The InC3 Study
Source: PLoS One. 2015 Apr 2;10(4):e0122232. doi: 10.1371/journal.pone.0122232 (PMC4383375; doi:10.1371/journal.pone.0122232)
Supplement: S1 Table — (DOC) [file pone.0122232.s003.doc]

**S1 Table. Characteristics of individuals with acute HCV infection who were included (well-characterized acute HCV) and were not included in further analysis of patterns of HCV RNA levels during acute infection**

|  | Included individuals (n=162)  n (%)^*^ | Excluded individuals (n=481)  n (%)^*^ | *P* |
| --- | --- | --- | --- |
| **Site** |  |  | <0.01 |
| ACS (the Netherlands) | 17 (10) | 27 (6) |  |
| ATAHC (Australia) | 6 (4) | 119 (25) |  |
| BAHSTION (United States) | 13 (8) | 37 (8) |  |
| BBAASH (United States) | 67 (41) | 47 (10) |  |
| HEPCO (Canada) | 2 (1) | 76 (16) |  |
| HITS-c (Australia) | 3 (2) | 7 (1) |  |
| HITS-p (Australia) | 12 (7) | 78 (16) |  |
| N2 (Australia) | 0 (0) | 17 (3) |  |
| UFO (United States) | 42 (26) | 73 (15) |  |
| **Median age at the time of HCV infection, yrs (IQR^**^)** | 24 (21-28) | 27 (23-34) | <0.01 |
| **Sex** |  |  | 0.16 |
| Female | 67 (41) | 163 (34) |  |
| Male | 94 (58) | 317 (66) |  |
| Unknown | 1 (1) | 1 (<1) |  |
| **Ethnicity** |  |  | 0.05 |
| Caucasian | 128 (79) | 397 (82) |  |
| Black | 11 (7) | 13 (3) |  |
| Indigenous | 4 (2) | 28 (6) |  |
| Other | 15 (9) | 32 (7) |  |
| Unknown | 4 (2) | 11 (2) |  |
| **History of injecting drug use** | 161 (99) | 455 (95) | <0.01 |
| **Clinical illness at the time of acute HCV infection** |  |  | <0.01 |
| No | 11 (7) | 109 (23) |  |
| Yes | 17 (10) | 122 (25) |  |
| Unknown | 134 (83) | 250 (52) |  |
| ***IFNL3* genotype (rs12979860)** |  |  | 0.51 |
| TT | 16 (10) | 48 (10) |  |
| CT | 61 (38) | 154 (32) |  |
| CC | 66 (41) | 206 (43) |  |
| Unknown | 19 (12) | 73 (15) |  |
| **HIV status at the time of HCV infection** |  |  | 0.09 |
| Negative | 151 (93) | 423 (88) |  |
| Positive | 5 (3) | 39 (8) |  |
| Unknown | 6 (4) | 19 (4) |  |
| **HCV genotype** |  |  | 0.33 |
| Genotype 1 | 88 (54) | 214 (44) |  |
| Genotype 2 | 9 (6) | 24 (5) |  |
| Genotype 3 | 41 (25) | 146 (30) |  |
| Genotype 4 | 1 (1) | 6 (1) |  |
| Genotype 6 | 0 (0) | 4 (1) |  |
| Mixed genotype | 2 (1) | 12 (2) |  |
| Unknown | 21 (13) | 75 (16) |  |

* Percentages indicate column percentages

** IQR: Inter-quartile range
